# Effects of Chinese traditional five-element music intervention on postoperative delirium and sleep quality in elderly patients after non-cardiac surgery: a randomized controlled trial

**DOI:** 10.1186/s13741-024-00408-5

**Published:** 2024-05-28

**Authors:** Shuang Han, Zenghua Cai, Longlu Cao, Jianli Li, Lining Huang

**Affiliations:** 1 Department of Anesthesiology, The Second Hospital of Hebei Medical University, Shijiazhuang, 050061 Hebei China; 2 Department of Anesthesiology, Hebei General Hospital, Shijiazhuang, 050051 Hebei China; 3 Department of Anesthesiology, Bethune International Peace Hospital, Shijiazhuang, 050082 Hebei China

**Keywords:** Music therapy, Medicine, Chinese traditional, Sleep disorders, Sleep measure, Delirium, Aged

## Abstract

**Background:**

Postoperative delirium (POD) is a common neurologic disorder among elderly patients after non-cardiac surgery, which leads to various negative outcomes. Sleep disorder is considered an important cause of POD. The objective of this study was to investigate whether the Chinese traditional five-element music intervention could reduce POD by improving sleep quality in elderly patients undergoing non-cardiac surgery.

**Methods:**

A total of 132 patients aged 65 to 90 years who underwent non-cardiac surgery were randomized to two groups: the intervention (*n* = 60) and the control group (*n* = 63). Patients in the intervention group were subjected to the Chinese traditional five-element music intervention during the perioperative, while patients in the control group had no music intervention. POD was evaluated using the Confusion Assessment Method (CAM) in the first 5 days after surgery. The Richards‒Campbell Sleep Questionnaire (RCSQ) was used to assess subjective sleep quality. The levels of nocturnal melatonin and cortisol in saliva were measured on the preoperative and the first 2 postoperative days.

**Results:**

The incidence of POD within 5 days was 27.0% in the control group and 11.7% in the intervention group. Preoperative PSQI and MMSE scores were associated with POD. The RCSQ scores on the first postoperative day were significantly decreased in the two groups compared to the preoperative day. Compared to the control group, the RCSQ scores showed a significant improvement in the intervention group on the first postoperative day. Compared to the control group, the level of saliva melatonin in the intervention group showed a significant increase on the first postoperative day. However, there was no statistical difference in cortisol levels between the two groups.

**Conclusions:**

Chinese traditional five-element music intervention decreased the incidence of POD in elderly patients who underwent noncardiac surgery via improving sleep quality, which may be associated with increased levels of melatonin.

## Introduction

Postoperative delirium (POD) is defined as an acute brain dysfunction characterized by disordered awareness, attention, cognition, and disturbances of the sleep–wake cycle (Li et al. 2022). POD commonly occurs 2–5 days after surgery, which could increase the incidence of postoperative complications, prolong postoperative recovery, and reduce patients’ quality of life (Inouye et al. 2014). Multiple mechanisms have been reported to be involved in the development of POD, including neurotransmitters, stress responses, and inflammatory and biological rhythms (Mulkey et al. 2018; Chen et al. 2022; Tan et al. 2020).

Moreover, recent studies have shown that sleep disorders are considered an important etiology of POD (Leung et al. 2015; Ulsa et al. 2022). Based on the international classification of sleep disorders (ICSD), types of sleep disorders include insomnia, sleep-related breathing disorders, circadian rhythm sleep–wake disorders, central disorders of hypersomnolence, parasomnias, sleep-related movement disorders, and other sleep disorders. Additionally, perioperative sleep disorders are prevalent in hospitalized individuals, often presenting with reduced sleep quality and duration, early awakening, frequent nightmares, sleep terrors, and sleep-related breathing disorders, which can impair brain function and increase the incidence of POD (Fadayomi et al. 2018). Therefore, it is essential to explore the appropriate strategies for improving sleep quality to prevent POD in elderly patients undergoing noncardiac surgery.

Multiple strategies have been used to optimize postoperative sleep in recent years. Many studies have confirmed the efficacy of nonpharmacologic interventions, including earplugs and eye masks, acupoint therapies, and listening to music, in postoperative patients (Leong et al. 2021; Wei et al. 2023; Kim et al. 2020). Music intervention, in particular, is a painless, safe, inexpensive, and practical non-drug treatment that has been widely used to improve sleep quality. A recent study demonstrated that a single interactive music therapy intervention improved short-term sleep quality in postoperative elderly patients (Kim et al. 2020). Moreover, a meta-analysis indicated an approximately 50% reduction in the risk of delirium after exposure to music compared to non-exposure in postsurgical and critically ill ICU patients (Golubovic et al. 2022). However, these studies were conducted mostly on postoperative patients and ICU patients. Furthermore, sleep quality and postoperative delirium were not assessed concurrently. Unlike other studies (Kukreja et al. 2020; Garcia Guerra et al. 2019), we assessed general anesthesia patients who listened to music during the perioperative (including 2 days before surgery, intraoperative and postoperative). Anesthesia is generally considered a state of insensibility, however, a recent systematic review and meta-analysis showed that auditory stimuli could be perceived under general anesthesia, leading to the formation of implicit memory without generating explicit consciousness (Fu et al. 2021). A recent study showed that playing therapeutic suggestions during general anesthesia could reduce postoperative pain and opioid use (Nowak et al. 2020). Therefore, the present study hypothesized that music may have beneficial effects on general anesthesia patients.

At present, the choice of music mainly includes classical music, pop music and some other types of music with soft tones and low dynamic amplitudes. “Chinese traditional five-element music intervention”, a unique nonpharmacological intervention, combines the strengths of traditional Chinese medicine theory and music therapy, which consists of five different styles of music with one main tone and respective characteristics (Zhang and Gao 2022). Recently, Chinese traditional five-element music intervention has been widely used in the clinic. A recent study showed that traditional Chinese five-element music could effectively reduce anxiety and depression in patients with cancer (Yang et al. 2021). Therefore, we hypothesized that listening to Chinese traditional five-element music during the perioperation could reduce the incidence of POD by improving the sleep quality of elderly patients who underwent noncardiac surgery. We performed a randomized controlled clinical trial (RCT) to investigate the effect of traditional Chinese five-element music intervention on POD in older adults after noncardiac surgery and its specific mechanism.

## Materials and methods

### Study design and participants

This prospective randomized controlled parallel-group clinical study was conducted at Hebei General Hospital in China and was approved by the Medical Ethics Committee of Hebei General Hospital. This study was registered in the Chinese Clinical Trials Registry (Chi-CTR-2200059088). Subjects in the research provided written informed consent prior to surgery. This study was conducted in accordance with the guidelines outlined in the Declaration of Helsinki.

Patients scheduled for noncardiac surgery were screened from January 2022 to October 2022. Inclusion criteria were as follows: aged 65 to 90 years; American Society of Anesthesiologists (ASA) grade II or III; scheduled to undergo elective noncardiac surgery lasting 1 h or longer under general anesthesia; and estimated length of stay ≥ 3 days. Patients with hearing or communication difficulties, psychosis, and refusal to participate were excluded. Patients were excluded due to canceled operations, unplanned transfer to the intensive care unit (ICU), or the requirement of mechanical ventilation after surgery.

### Procedures

Our study used sequentially numbered, opaque closed envelopes to randomize patients to either the intervention group or the control group. A randomization sequence by permuted blocks of 4 and 6 with an allocation of 1:1 was generated by a computer program and concealment was maintained by an independent investigator with no clinical involvement in the trial.

Two days before surgery, Bluetooth headsets and an MP3 player were provided. Patients were educated on the use of the devices during recruitment and encouraged to wear the headsets to listen to music for 30 min between 9:00 and 10:00 pm randomly. In addition, the music was played randomly on a loop during the surgery and the period of recovery from anesthesia, with standard volume for 20 min, followed by 10 min of silence. The music was selected from the Chinese traditional five-element music positive mode (ISBN:7–88,032-383-X) published by the Chinese Medical Electronic Audio and Video Society. The control group wore headphones during the surgery to shield them from voices and noises in the operating room. The medical staff involved with patient care (anesthetist, nurse) and the outcome evaluators were blind to randomization status.

### Intervention fidelity

To ensure intervention fidelity several strategies were used. To ensure patients receive an equal dose of intervention, patients listen to music for 30 min between 9:00 and 10:00 pm 2 days before surgery under the supervision of a nurse who worked overnight. During the procedures, the intervention providers put on headphones for the patient and ensured that the music was playing normally. The intervention was conducted by a resident anesthesiologist who was not involved in the data analysis or the anesthetic management and received comprehensive guidance and equipment use training. Firstly, the intervention implementers were informed of the objectives of the training to ensure its effectiveness. Secondly, clearly inform the intervention time, place, and the use method of devices to ensure that the intervention is comprehensive and systematic.

### Anesthesia method

All patients underwent regular monitoring of ECG, arterial pressure, and pulse oxygen saturation. Anesthesia induction was performed with sufentanil 0.3 ~ 0.5 μg/kg, etomidate 0.3 mg/kg, and rocuronium 0.9 mg/kg. Anesthesia was maintained with remifentanil, propofol, and cisatracurium to maintain BIS values between 40 and 60. Participants received patient-controlled intravenous analgesia (PCIA) or intravenous infusion analgesics after surgery. Intraoperative hypotension was defined as systolic blood pressure < 90 mmHg or MAP < 65 mmHg. Hypotension was managed by controlling volume status and administering 3–6 mg of ephedrine or 5–10 mcg of norepinephrine.

### Outcome assessment

All participants underwent the Chinese version of the confusion assessment method (CAM) screening for postoperative delirium twice daily until 5 days after surgery (in the morning from 08:00 to 10:00 am and in the evening from 6:00 to 8:00 pm). A patient who met the CAM criteria for delirium at least once on the five postoperative days was considered to have POD. The diagnosis of delirium was based on established criteria according to the CAM (Inouye et al. 1990) assessments. CAM is the most frequently used method for delirium testing worldwide and is based on the presence of four essential delirium signs: (1) altered level of consciousness, (2) acute onset and varying pattern of symptoms, (3) disorganized thinking, and (4) inattentive. Delirium was defined as the presence of (1) and (2), accompanied by (3) or (4) or both. This study included strict standardization and training procedures for assessing delirium.

To assess the quality of sleep 1 month before the operation, the patients completed the Chinese version of the Pittsburgh Sleep Quality Index questionnaire (PSQI) (Smyth 1999). The test consists of eighteen items divided into seven dimensions: subjective sleep quality, duration, efficiency, hypnotic drugs, sleep disturbance, and daytime dysfunction. The components vary in score from 0 to 3, and the total score can be anywhere between 0 and 21. A higher score indicates more severe sleep disturbance. A cutoff of the score at > 5 indicates that patients have sleep disturbances.

All participants completed the Chinese version of the Richards‒Campbell Sleep Questionnaire (RCSQ) 1 day before surgery and 1 to 2 days after surgery to assess sleep quality and duration. Each of the five items on the questionnaire possesses a visual scale from 0 (the worst sleep) to 100 (the best sleep quality), and the participant describes their impression of their sleep during this time. The domains include subjective sleep quality, depth of sleep, ease of falling asleep, frequency of awakenings, and ease of going back to sleep. The average of five factors is considered to determine the overall quality of sleep (Kamdar et al. 2012).

The Chinese version of the Mini-Mental State Examination (MMSE), which evaluates orientation, language, memory, attention, and spatial skills, was conducted on the day before the operation to examine the cognitive function of participants. The total score of the MMSE ranges from 0 to 30, with a score of 24 or greater as no cognitive impairment. We evaluated the associations of the preoperative MMSE scores with POD.

To measure cortisol and melatonin concentrations, saliva was collected at 10:00 pm on the day prior to surgery and 1–2 days after surgery. Prior to sampling, participants were instructed to perform oral cleaning and to refrain from eating, smoking, and chewing gum for 30 min. Saliva (2–3 mL) was collected and slowly injected into a sterile centrifuge tube, which was then centrifuged at 3000 × *g* for 20 min. The supernatant was separated into a 1.5-mL aseptic centrifuge tube and frozen at − 80 °C for laboratory analysis. The concentrations of salivary melatonin and cortisol were detected by ELISA (Salivary Cortisol ELISA Kit and Salivary Melatonin ELISA Kit). All assays were performed according to the manufacturer’s protocols.

### Statistical analysis

The sample size calculation was primarily based on the incidence of POD. Previous studies reported that 25% of patients who underwent noncardiac surgery experienced POD (Ziman et al. 2020). We made the conservative assumption that the incidence of POD in the intervention group would be reduced by 50% in accordance with previously reported research (Burton et al. 2021). Using a two-sided α value of 0.05% and 80% power, the sample size needed to detect differences was calculated to be 118 patients using Gpower3.1 software. With a 10% dropout rate in mind, we intended to enroll 132 patients in this research. The Kolmogorov–Smirnov test was conducted to check data normality. Count data are presented as ratios based on the type of variable and distribution, while measurement data are shown as the means ± standard deviations, medians, and interquartile ranges (IQRs). The groups were compared using the independent samples *t*-test or nonparametric Wilcoxon rank sum test, and the count data were compared using the chi-square (*χ*2) test. To compare the levels of cortisol and melatonin at different times, we employed a two-way repeated measures analysis of variance (ANOVA).

Additionally, the arguments with *P* value < 0.1 by univariate regression analysis were performed a forward stepwise multivariate logistic regression analysis, thus controlling the confounding bias and screening out related factors for POD. For the incidence of POD, odds ratios (ORs) and 95% confidence intervals (CIs) were computed. An alpha of 0.05 was considered significant. SPSS version 23.0 (SPSS Inc., Chicago, IL, USA) was used to analyze the data.

## Results

**Fig. 1:**
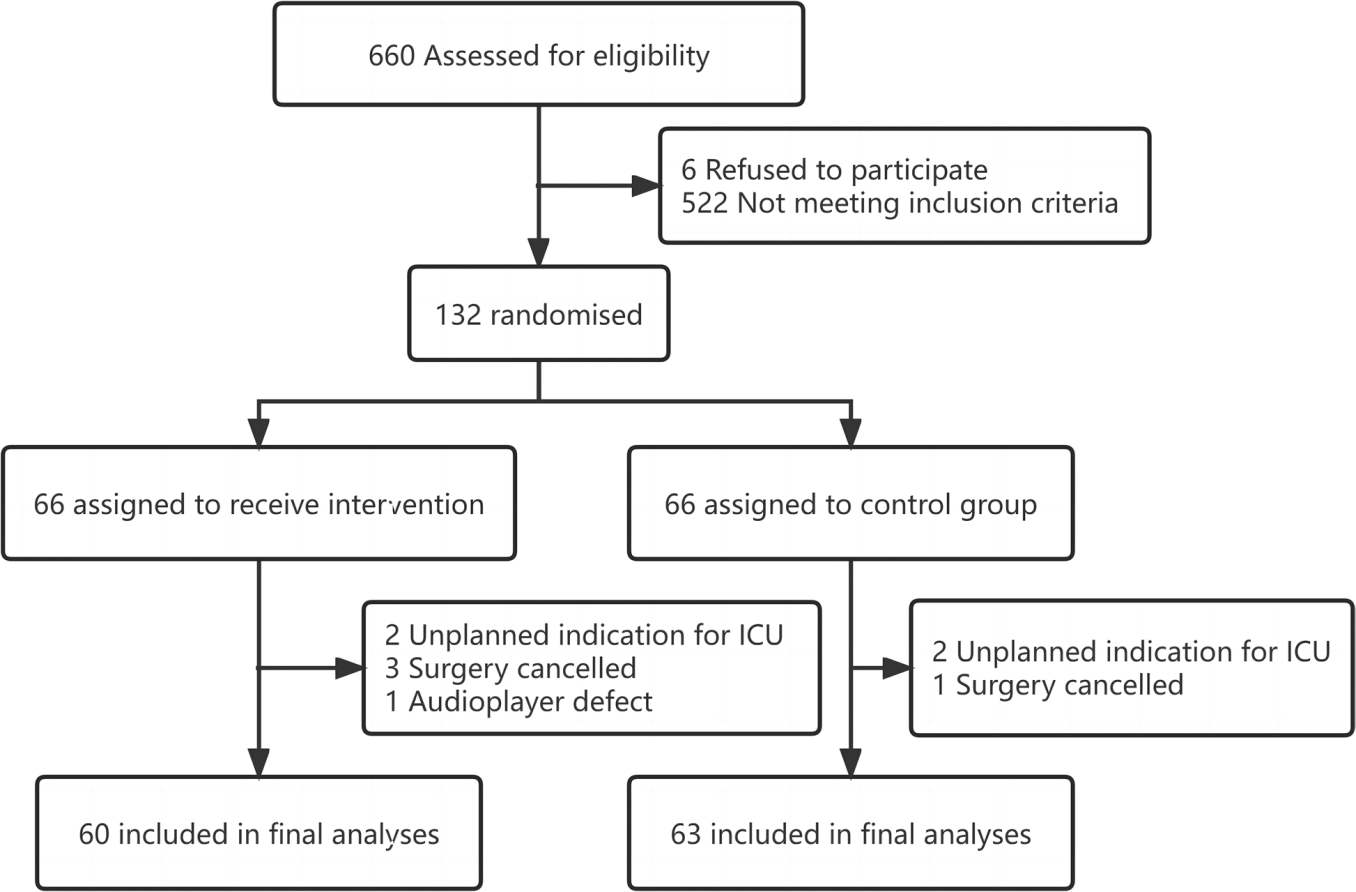
Study flow diagram

From January 11, 2022, to October 31, 2022, a total of 660 patients were screened for study participation. Among these patients, 132 were enrolled in the study and randomly assigned to the intervention group (*n* = 66) or the control group (*n* = 66). In the intervention group, six patients were withdrawn due to unplanned indications for ICU stay (*n* = 2), surgery cancellation (*n* = 3), and audio player defects (*n* = 1). In the control group, two patients were transferred to the ICU, and one patient’s surgery was canceled. Ultimately, 123 participants completed the study, and data analyses were performed on 63 of the cases in the control group and 60 cases within the intervention group (Fig. 1).

### Baseline data

The demographic analysis results of the patients are presented in Table 1. Out of 123 participants, the minimum age was 65 years, and the maximum age was 87 years. No significant difference was reported in terms of sex, age, duration of surgery, duration of anesthesia, type of surgery, ACCI scores, RCSQ scores, MMSE scores, PSQI scores, intraoperative hypotension, and length of hospital stay between the two groups (*P* > 0.05). Overall, the two groups were well-matched for baseline and perioperative variables.

**Table 1:** Baseline characteristics of participants

Characteristics	Intervention group (*n* = 60)	Control group (*n* = 63)	*P* value
Age (years), mean (SD)	73.3 (5.4)	73.3 (5.6)	0.971
Sex			0.862
Male	39 (65.0)	40 (63.5)	–
Female	21 (35.0)	23 (36.5)	–
BMI, mean (SD), kg/m^2^	24.5 (3.1)	25.1 (2.6)	0.306
ASA fitness grade			0.844
II	19 (31.7)	21 (33.3)	–
III	41 (68.3)	42 (66.7)	–
Education			0.365
Illiterate	8 (13.3)	7 (11.1)	–
Primary school	23 (38.3)	19 (30.2)	–
Junior high school	17 (28.3)	27 (42.9)	–
Senior high school	10 (16.7)	6 (9.5)	–
University or higher	2 (3.3)	4 (6.3)	–
Preoperative co-morbidity (yes, %)			
Hypertension	26 (43.3)	30 (47.6)	0.633
Diabetes mellitus	10 (16.7)	15 (23.8)	0.325
Cardiovascular disease	16 (26.7)	14 (22.2)	0.566
Type of surgery			0.948
Gastrointestinal	38 (63.3)	36 (57.1)	–
Hepatobiliary	8 (13.3)	7 (11.1)	–
Urological	8 (13.3)	13 (20.6)	–
Orthopaedic or spine	3 (5)	3 (4.8)	–
Gynecological	1 (1.7)	1 (1.6)	–
Thoracic	1 (1.7)	2 (3.2)	–
Vascular	1 (1.7)	1 (1.6)	–
ACCI scores median (interquartile range)	7 (5, 8)	6 (5, 7)	0.185
Pre-op PSQI scores median (interquartile range)	5 (4.25, 8.75)	5 (4, 8)	0.917
Pre-op RCSQ scores, median (interquartile range)	70 (57, 75)	68 (57, 76)	1.000
Pre-op sleep disturbances^a^	17(28.3)	17(27)	0.867
Pre-op MMSE scores, median (interquartile range)	27 (25, 28)	26 (24, 28)	0.361
Intraoperative medication			
Propofol does (mg), mean (SD)	925 (433)	921 (401)	0.965
Remifentanil does (mg), median (interquartile range)	1.59 (1.11, 2.75)	1.86 (1.27, 2.37)	0.719
Intraoperative hypotension (yes, %)	2 (3.3)	6 (9.5)	0.305
Duration of anesthesia (min), mean (SD)	214.8 (77.9)	210.1 (74.0)	0.736
Duration of surgery (min), mean (SD)	157.5 (112.75, 225.25)	160 (112, 200)	0.640
Length of stay in hospital (days) median (interquartile range)	14.5 (11, 21)	15 (11, 18)	0.905
ALB (g/L), mean (SD)	37.1 (4.4)	35.6 (5.3)	0.101
Hb (g/L), mean (SD)	121.7 (20.3)	120.1 (24.5)	0.692

Data are number (%), mean (SD), or median (interquartile range)

*Abbreviations*: *BMI* body mass index, *SD* standard deviation, *COPD* chronic obstructive pulmonary disease, *ACCI* age-adjusted Charlson Comorbidity Index, *PSQI* Pittsburgh sleep quality index, *RCSQ* Richards-Campbell Sleep Questionnaire, *MMSE* Mini-Mental State Examination, *ABL* albumin, *Hb* hemoglobin

^a^Pre-op sleep disturbances are PSQI score > 5

### The incidence of POD

Postoperative delirium occurred in 17 (27%) of 63 patients in the control group and in 7 (11.7%) of 60 patients in the intervention group over postoperative days 1–5. For the primary outcome of the study, the POD incidence was considerably lower in the intervention group than in the control group (*P* = 0.032). In addition, when patients were stratified according to whether they had sleep disturbances before surgery, the incidence of POD in the preoperative sleep disturbances patients who listened to music was reduced(*P* = 0.005) (Table 2).

**Table 2:** Incidence of delirium between groups

	Intervention group (*n* = 60)	Control group (*n* = 63)	*P* value
Overall incidence of delirium^a^ *n* (%)	7 (11.7)	17 (27.0)	0.032
Incidence of delirium according to preoperative sleep condition^b^, *n* (%)			
Pre-op sleep disturbances	3 (17.6%) (*n* = 17)	11 (64.7%) (*n* = 17)	0.005
No Pre-op sleep disturbances	4 (9.3%) (*n* = 43)	6 (13.9%) (*n* = 46)	0.577

^a^Occurrence of delirium at any time during the first 5 days after surgery

^b^Stratified according to whether the presence of pre-op sleep disturbances

### Sleep quality

The RCSQ was employed to evaluate sleep quality on the first 2 days after surgery. Sleep quality evaluated with RCSQ on the first postoperative day was significantly improved in the intervention group than in the control group (Table 3). In comparison to the control group, the RCSQ scores in the intervention group were significantly increased on the first postoperative day [54 (43.3, 68) vs. 48 (43, 56), *P* = 0.017]. However, there was no difference in the efficacy of Chinese traditional five-element music in improving patients’ sleep quality on the second postoperative day.

**Table 3:** Comparison of RCSQ scores between groups. Values are median (IQR [range])

RCSQ scores	Intervention group (*n* = 60)	Control group (*n* = 63)	*F*	*P* value
Preoperative night	70 (57, 75)	68 (57, 76)	0.018	0.894
POD1	54 (43.3, 68)	48 (43, 56)	5.905	0.017*
POD2	64.5 (50.3, 71.8)	63 (56, 69)	0.206	0.651

^*^ < 0.05 was considered statistically significant

*Abbreviations*: *RCSQ* Richards-Campbell Sleep Questionnaire, *POD1* postoperative day 1, *POD2* postoperative day 2

### Melatonin and cortisol in saliva

**Fig. 2:**
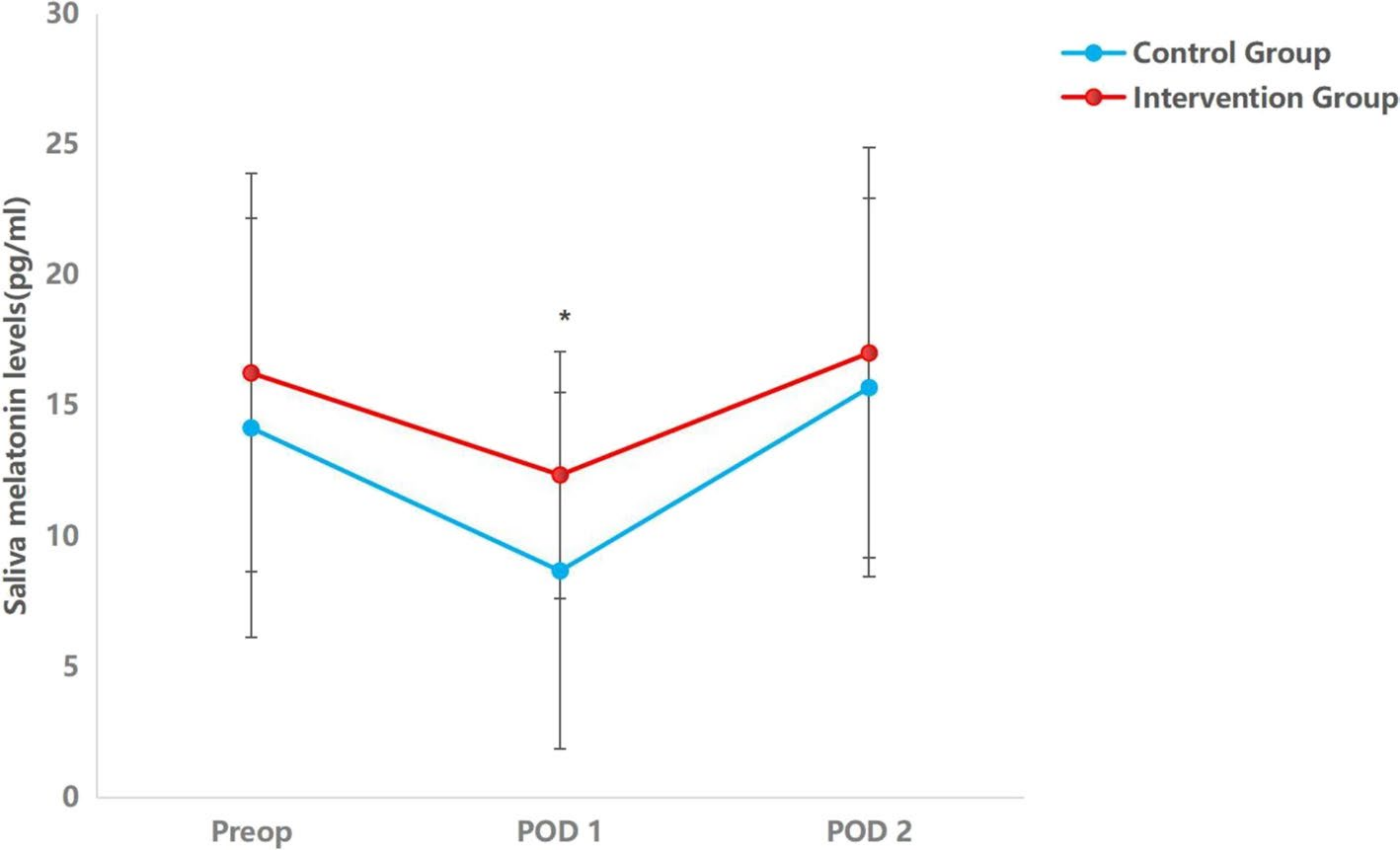
The trend of melatonin saliva levels in groups of intervention and control at three measurement points. The within-group comparison shows that the saliva levels of melatonin decreased on postoperative day 1 in both groups (*P* = 0.00). *Indicates a significant time interaction effect. *P < 0.05. Preop, preoperative day; POD1, postoperative day 1; POD2, postoperative day 2

**Fig. 3:**
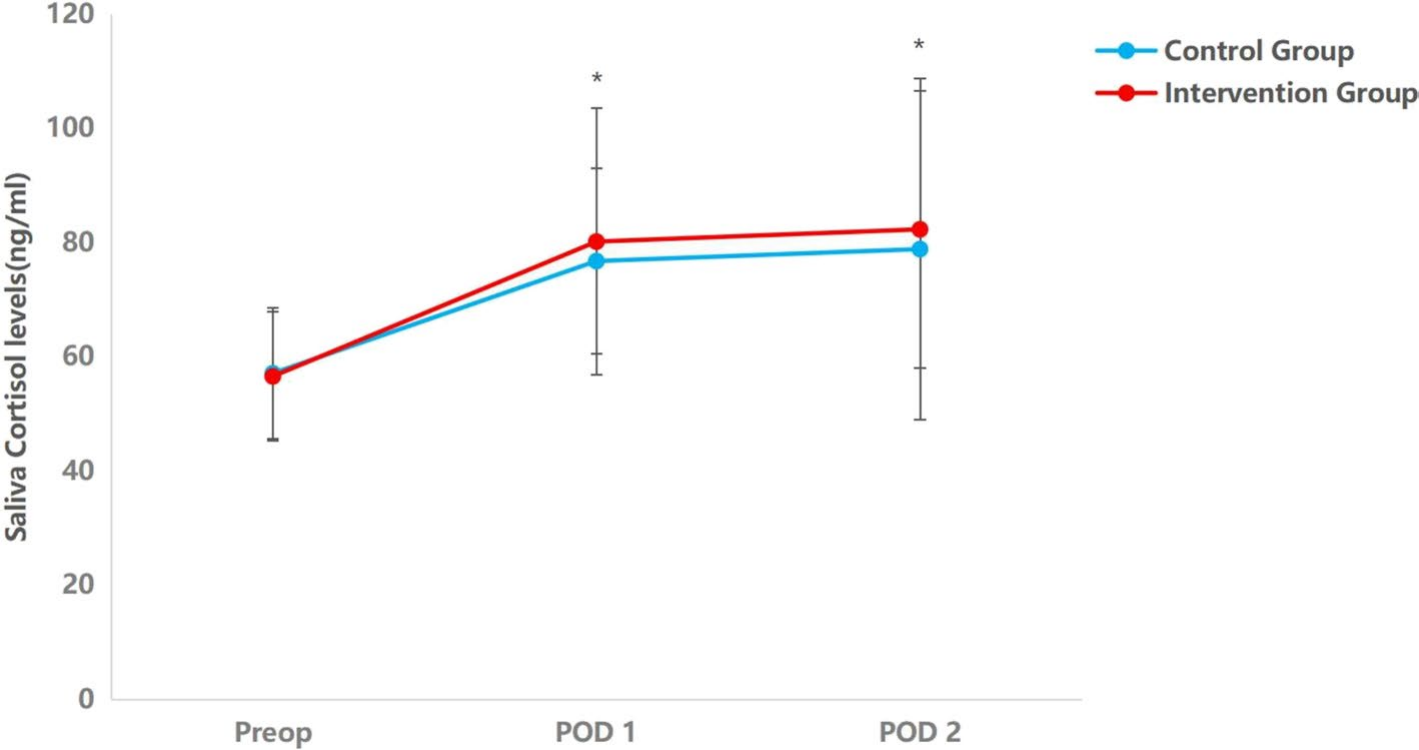
The trend of melatonin cortisol levels in groups of intervention and control at three measurement points. The within-group comparison shows that the cortisol levels on the first and second postoperative days were significantly higher than those on the night before surgery (*P* = 0.00). *Indicates a significant time interaction effect. *P < 0.05. POD1, postoperative day 1; POD2, postoperative day 2

Compared to the night before surgery, the melatonin levels were dramatically reduced on the first postoperative night. On the first postoperative day, the saliva melatonin levels of the intervention group were higher than those of the control group. The saliva cortisol levels of the first two postoperative nights were considerably higher compared to the day prior to surgery. However, no significant differences in cortisol levels were detected between the two groups (Table 4, Figs. 2 and 3).

**Table 4:** Saliva melatonin and cortisol levels between groups

	Intervention group (*n* = 60)	Control group (*n* = 63)	*F*	*P* value
Melatonin (pg/ml), mean (SD)				
Preoperative night	16.257 (8.102)	14.152 (7.629)	2.203	0.14
POD1	12.349 (6.881)	8.674 (4.724)	12.023	0.001*
POD2	17.023 (7.306)	15.696 (7.858)	0.939	0.334
Cortisol (ng/ml), mean (SD)				
Preoperative night	56.607 (11.344)	57.150 (11.493)	0.069	0.793
POD1	80.208 (23.517)	76.801 (16.209)	0.882	0.350
POD2	82.356 (24.467)	78.898 (29.954)	0.489	0.486

^*^ < 0.05 was considered statistically significant

*Abbreviations*: *POD1* postoperative day 1, *POD2* postoperative day 2

### Binary logistic regression on incidence of postoperative delirium

Univariate regression analysis showed that the *P* values of group allocation, preoperative PSQI scores, and preoperative MMSE scores are less than 0.1. Incorporating these factors into the multivariate logistic regression analysis, we found that music intervention showed significant statistical differences (adjusted odds ratio, 0.329; 95% CI, 0.110–0.988; *P* = 0.048). Furthermore, higher preoperative PSQI scores (adjusted odds ratio, 1.301; 95% CI, 1.132–1.494; *P* = 0.000) and lower baseline MMSE scores (adjusted odds ratio, 0.839; 95% CI, 0.719–0.979; *P* = 0.026) were associated with a higher incidence of POD. Age, gender, preoperative Hb concentration, and intraoperative hypotension were not associated with POD (Table 5).

**Table 5:** Univariate and multivariate logistic regression analyses on the incidence of postoperative delirium

Variables	Univariate		Multivariate	
	Unadjusted OR (95% CI)	*P* value	Adjusted OR (95% CI)	*P* value
Age	0.953(0.879–1.034)	0.244	–	–
Male sex (reference, female)	0.875(0.341–2.245)	0.781	–	–
Hb	0.988(0.968–1.007)	0.213	–	–
Intraoperative hypotension(Yes vs. No)	0.710(0.134–3.756)	0.687	–	–
Group allocation (intervention v control)	0.357(0.136–0.938)	**0.037**	0.329(0.110–0.988)	0.048
Pre-op MMSE scores	0.806(0.701–0.927)	**0.003**	0.839(0.719–0.979)	0.026
Pre-op PSQI scores	1.311(1.154–1.490)	**0.000**	1.301(1.132–1.494)	0.000

*Abbreviations*: *Hb* hemoglobin, *MMSE* Mini-Mental State Examination, *PSQI* Pittsburgh sleep quality index, *OR* odds ratio

ORs are reported for logistic regression analyses with 95% CIs. Dashes indicate data not applicable

## Discussion

In our study, compared to the control group, a 15.3% reduction in POD incidence in the intervention group was clinically meaningful. Our results suggested that Chinese traditional five-element music intervention decreased the incidence of POD in elderly patients who underwent noncardiac surgery via improving sleep quality, which may be associated with increased levels of melatonin. Binary logistic regression analysis showed that the intervention therapy was an independent factor affecting postoperative delirium. In addition, preoperative sleep quality and MMSE score affected postoperative delirium factors. Our study provides a reasonable basis for preventing POD in elderly patients with traditional Chinese five-element music intervention who underwent noncardiac surgery.

The present study revealed a 27% incidence of POD among patients in the control group, which was similar to some previous studies (Ziman et al. 2020; Su et al. 2016). Moreover, the incidence of POD varied by surgical type. A study showed that the incidence of POD in cardiac, orthopedic, general, vascular, and urological surgery was 23.6%, 15.2%, 26.6%, 15.8%, and 12.7%, respectively (Watt et al. 2018), especially in geriatric patients undergoing surgery with hip fracture, where the prevalence can reach as high as around 50% (Jeon and Sohng 2021). Therefore, POD, a common and major complication after surgery, is associated with increased mortality. It is essential to explore the specific mechanism of POD and the appropriate strategies to prevent POD in the elderly following noncardiac surgery.

In recent years, studies have suggested that sleep disorders are an important causal factor of POD (Fadayomi et al. 2018). There is a close association between POD and sleep, in many POD patients, often accompanied by sleep–wake cycle disorders. Studies have reported that 42% of patients developed postoperative sleep disorders, while 24% of patients needed medication for that (Kain and Caldwell-Andrews 2003). Similarly, the outcomes of our study showed that RCSQ scores were markedly lower on the first postoperative day than before the operation, which suggested that the patient’s sleep quality decreased after the operation. Additionally, numerous studies have demonstrated that preoperative sleep disturbances are recognized as an independent risk factor for POD, which could increase the incidence of POD in geriatric patients and negatively impact postoperative rapid recovery (Wang et al. 2020; Todd et al. 2017). According to whether patients had preoperative sleep disorders, a post hoc subgroup analysis was conducted, and the results showed that patients with sleep disturbance before surgery had an increased incidence of POD, which was consistent with a previous study (Wang et al. 2020). Additionally, Age and gender have been verified to be closely associated with sleep quality and POD (Wang et al. 2022). Therefore, we included age and gender as potential confounders in the model and adjusted for these factors in our analyses. After adjusting related factors by multivariate logistic regression analysis, our result showed that preoperative PSQI scores were associated with the incidence of POD. A positive relationship between higher pre-operative PSQI scores (i.e., worse sleep quality) and odds of POD,that is, patients with poor preoperative sleep quality had a high incidence of POD. Therefore, we hypothesized that perioperative sleep quality was related to POD, enhancing the sleep quality in geriatric patients is essential to prevent and treat POD.

MMSE has been widely regarded as a simple scale to evaluate cognitive function, and lower scores indicated poorer cognitive function (Segernäs et al. 2022). Our results showed that MMSE scores were negatively associated with odds of POD, which suggested that patients with low cognition function would increase the probability of developing POD. These results were consistent with those from previous studies showing that postoperative delirium was related to a decrease in preoperative cognitive reserve (Adogwa et al. 2018; Chen et al. 2022). This suggests that POD is mostly caused by an aging brain. Mounting evidence points out that the sleep–wake cycle plays a crucial part in brain aging. Thus, more effective perioperative management strategies are necessary for these poorer cognitive function patients with sleep disorders.

At present, nonpharmacological therapies for improving sleep quality include listening to music, covering sensory input (such as wearing earplugs or eye masks), creating a quiet environment, and acupoint therapies. Music intervention, as a painless, safe, inexpensive, and practical nondrug treatment, could significantly improve insomnia and anxiety (Umbrello et al. 2019; Kavurmaci et al. 2020). Recent reviews and meta-analyses corroborated the effectiveness of music intervention in reducing anxiety and pain in patients undergoing surgery (Hole et al. 2015; Kühlmann et al. 2018). Additional evidence suggests that music listening is an effective intervention to reduce POD in elderly individuals undergoing selective hip and knee surgery (Golubovic et al. 2022) and improve the postoperative sleep quality of elderly patients in the ICU (Kim et al. 2020). At present, the choice of music mainly includes classical music, pop music, and other music with soft tones and low dynamic amplitudes. Chinese traditional five-element music intervention based on the five-element theory treats diseases by using five different music tunes, such as Gong, Shang, Jue, Zhi, and Yu, which connect to the liver, heart, spleen, lung, and kidney, respectively. Recent research has shown that traditional Chinese five-element music could effectively reduce anxiety and depression in cancer patients (Liao et al. 2018) and pregnant women (Wu et al. 2020). Our study demonstrated that five-element music intervention could improve postoperative sleep quality and reduce the incidence of POD in elderly patients undergoing noncardiac surgery. Additionally, binary logistic regression analysis showed that five-element music treatment has prophylactic efficacy in the incidence of POD. Therefore, Chinese traditional five-element music intervention could improve the sleep quality of elderly patients and reduce the incidence of POD.

However, the specific mechanisms of improving sleep quality by music exerting its effects are unknown. A large systematic review found that music intervention could have an impact on various neurotransmitters, cytokines, and hormones (Fancourt et al. 2014). Melatonin is produced by the pineal gland, while cortisol is one of the main glucocorticoid hormones released by the adrenal cortex, their secretion has a circadian rhythm and plays a role in regulating the sleep–wake cycle. Moreover, Multiple studies have shown that sleep disturbance, such as insomnia, was associated with increased ACTH and cortisol secretion (Vgontzas and Chrousos 2002). Melatonin and cortisol levels are significantly associated with the risk of delirium in patients (Sun et al. 2021; Song et al. 2021; Scholtens et al. 2017). A recent animal experiment reported that melatonin intervention could be a potential preventative approach for postoperative sleep disorder and delirium (Jia et al. 2021). A study showed that interactive music therapy increased salivary melatonin levels and improved sleep quality in postoperative elderly patients (Kim et al. 2020). These studies suggest that Melatonin and cortisol levels were associated with POD and sleep disorders. We observed a decline in saliva melatonin levels and an increase in cortisol levels on the first postoperative day, which might be the cause of decreased sleep quality. Compared with the control group, the incidence of POD decreased, and melatonin levels and RCSQ scores increased on the first day after surgery in the intervention group,however, the difference in cortisol levels was not statistically significant. These results suggested that the Chinese traditional five-element music intervention decreased the incidence of POD in elderly patients who underwent noncardiac surgery via improving sleep quality, which may be associated with increased levels of melatonin. However, cortisol secretion could be affected by many factors, such as disease, surgical intervention, anesthesia, and medication, possibly masking the potential impact of the intervention.

Our study has some limitations that should be discussed. First, we used the RCSQ as a means to assess the sleep quality of patients, which is a subjective measure of sleep quality. Second, the contribution of factors other than the music intervention remains unclear, for example, the beneficial effect of earphones blocking out ambient noise cannot be ruled out. Moreover, nighttime light exposure might impact melatonin secretion; however, we did not specify light levels in the ward when we collected the saliva samples, thus there are limitations to interpreting the results.

## Conclusions

In conclusion, our results demonstrate that Chinese traditional five-element music intervention decreased the incidence of POD in elderly patients who underwent noncardiac surgery via improving sleep quality, which may be associated with increased levels of melatonin. Further research is needed to measure the mechanism of the positive effects of music intervention on humans and animals.

## Data Availability

The datasets used and/or analyzed during the current study are available from the corresponding author on reasonable request.
